# Development and Validation of a Novel and Fast Detection Method for *Cannabis sativa*: A 19-Plex Short Tandem Repeat Typing System

**DOI:** 10.3389/fpls.2022.837945

**Published:** 2022-02-28

**Authors:** Ruocheng Xia, Ruiyang Tao, Yiling Qu, Xiaochun Zhang, Huan Yu, Chunyan Yuan, Suhua Zhang, Chengtao Li

**Affiliations:** ^1^Academy of Forensic Sciences, Ministry of Justice, Shanghai, China; ^2^School of Forensic Medicine, Shanxi Medical University, Taiyuan, China; ^3^Department of Forensic Medicine, Inner Mongolia Medical University, Hohhot, China

**Keywords:** *Cannabis sativa*, short tandem repeats (STRs), polymerase chain reaction (PCR), capillary electrophoresis (CE), multiplex system, developmental validation

## Abstract

In recent years, influenced by the legalization of *Cannabis sativa* in some countries and regions, the number of people who smoke or abuse *C. sativa* has continuously grown, cases of transnational *C. sativa* trafficking have also been increasing. Therefore, fast and accurate identification and source tracking of *C. sativa* have become urgent social needs. In this study, we developed a new 19-plex short tandem repeats (STRs) typing system for *C. sativa*, which includes 15 autosomal STRs (D02-CANN1, C11-CANN1, 4910, B01-CANN1, E07-CANN1, 9269, B05-CANN1, H06-CANN2, 5159, nH09, CS1, ANUCS 305, 3735, and ANUCS 302 and 9043), two X-chromosome STRs (ANUCS 501 and 1528), one sex-determining marker (DM016, on Y-chromosome), and a quality control marker (DM029, on autosome). The whole polymerase chain reaction (PCR) process could finish within 1 h, making the system suitable for fast detection. The PCR products were detected and separated with an Applied Biosystems 3500*XL* Genetic Analyser. Developmental validation studies indicated that the 19-plex typing system was accurate, reliable and sensitive, which could also deconvolute mixed *C. sativa* samples. Specifically, the sensitivity study showed that a full genotyping profile was obtainable with as low as 125 pg of *C. sativa* DNA. The species specificity study demonstrated that this multiplex has no cross-reactivity with common non *C. sativa* DNA. In the population study, a total of 162 alleles at 15 autosomal STRs and 14 alleles at two X-chromosome STRs were detected among 85 samples. The efficiency parameters, including the total discrimination power (TDP) and the combined power of exclusion (CPE) of the system, were calculated to exceed 0.999 999 999 999 988 and 0.998 455 889 684 078, respectively, further proving that the system could meet the needs of individual identification. To the extent of the known studies, this is the first study that included the *C. sativa* sex-determining marker. In conclusion, the developed new 19-plex STR typing system can successfully achieve the purposes of species identification, gender determination, and individual identification, which could be a powerful tool in tracing trade routes of particular drug syndicates or dealers or in linking certain *C. sativa* to a crime scene.

## Introduction

*Cannabis sativa* is an annual dioecious herb belonging to the *Cannabis* genus of Cannabaceae. *C. sativa* is cultivated as an important cash crop in many countries. However, *C. sativa* has also been listed as one of the top three drugs alongside heroin and cocaine by the United Nations Convention on Drug Control due to tetrahydrocannabinal (THC) which is rich in flowers and leaves and is a highly addictive and narcotic hallucinogenic ingredient (Kohnemann et al., 2012). As the most widely cultivated, produced, trafficked and consumed drug in the world (Soorni et al., 2017). *C. sativa* is a diploid plant with 20 chromosomes (18 autosomal chromosomes and 2 sex chromosomes), with the chromosomal pattern of XX for female plants and XY for male plants (Bakel et al., 2011). The stems and leaves of male plants do not contain or contain extremely little THC, while the content of THC in female plants is relatively high (Matthew-Simmons et al., 2011). Therefore, female plants have medicinal value but also abuse potential.

In recent years, under the influence of legalization of *C. sativa* in Canada, Netherlands and some states in the United States, the number of people who smoke or abuse *C. Sativa* has continuously grown, with increasing cases of transnational *C. sativa* trafficking (Hillmer et al., 2020; Scheim et al., 2020). Therefore, fast and accurate judicial identification and source tracking of *C. sativa* have become an urgent social need to launch a scathing attack on drug crime. Conventionally, gas chromatography/mass spectrometry (GC/MS) is mostly applied for *C. sativa* identification, detecting whether biological samples contain THC (Taylor and Birkett, 2020). GC/MS could provide enough evidence for prosecuting a marijuana-possessing individual, while regarding the origin or individualization of the plant, few clues could be acquired by the test. In contrast, the genetic individualization of *C. sativa* plant raises the possibility of establishing relationships between different cases and assessing their belonging to a trade network, which would play key roles in illegal trade investigations.

The DNA method appears to have higher resolution for the individualization of *C. sativa* plants compared to the other techniques (Gilmore et al., 2006). Since 1990s, various DNA markers were developed and evaluated for the purpose of *C. sativa* identification, including random amplified polymorphic DNA (RAPD), sequence characterized amplified region (SCAR), DNA barcoding, short tandem repeats (STRs) and single nucleotide polymorphisms (SNPs) (Hsieh et al., 2003; Techen et al., 2010; Rotherham and Harbison, 2011; Mello et al., 2016; Soorni et al., 2017; Roman et al., 2019). PARD and SCAR genetic markers can identify only the species and sex of *C. sativa*, and DNA barcoding can accurately identify *C. sativa* from its adulterants, while none of them can be used for *C. sativa* individualization or origin inference. SNPs was able to differentiate between *C. sativa* and other species and to assess the genetic diversity of *C. sativa* (Rotherham and Harbison, 2011; Soorni et al., 2017), however, SNPs are bi-allele makers that show less polymorphism and the detection technologies for SNPs (e.g., next-generation sequencing and SNP Chip) consume high cost. Notably, STR [also known as simple sequence repeat (SSR)] is an oligonucleotide sequence composed of a core sequence of 2–6 bp in tandem because it has high sensitivity, high discrimination ability, species specificity, and accuracy and facilitates standardization. Based on the commonly used capillary electrophoresis (CE) platform, which is also cost saving, STRs have been widely utilized in individual identification, kinship analysis and group investigation for humans, animals and plants (Chakraborty et al., 1999; Tagliaro et al., 2010; Ramadan et al., 2018; Alhariri et al., 2021).

As a step toward understanding STR markers of *C. sativa*, emerging studies have tried to apply STRs for the investigation of *C. sativa*. Since 2003, several lines of new markers for *C. sativa* have been evaluated and optimized for forensic purposes (Alghanim and Almirall, 2003; Hsieh et al., 2003; Howard et al., 2008, 2009). At present, Houston et al. (2016, 2017) have developed two multiplex panels containing 13-STR markers, according to the International Society of Forensic Genetics (ISFG) and the Scientific Working Group on DNA Analysis Methods (SWGDAM) guidelines, which are also the most widely used panels for *C. sativa*. However, these two panels have been evaluated by Fett et al. (2019) and Ribeiro et al. (2020), which showed locus dropout and relatively low efficiency according to the analysis of population genetics. In addition, although it is very important to distinguish the sex of *C. sativa* (since differences exist in the THC content of *C. sativa* plants of different sexes), neither of the two panels could meet the needs.

To establish an accurate and fast detection system for *C. sativa*, and to improve the efficiency of individual identification, kinship testing and other research purposes for *C. sativa*, in this paper, a number of STR loci with good discriminative ability were selected, and one sex-determining marker and one quality control marker were also included. Based on a quick PCR amplification process, a reliable STR multiplex system for *C. sativa* was developed and optimized. Further developmental validation studies of the new multiplex were performed following guidelines established by SWGDAM and ISFG, including PCR conditions (annealing temperatures and cycling numbers), precision, accuracy, sensitivity, species specificity, stutter percentage, balance and statistical analysis. This system aims to provide a fast and effective tool that facilitates the police in tracing back trade routes of drug syndicates or dealers and linking different *C. sativa* plants to a crime scene.

## Materials and Methods

### Sample Collection and DNA Extraction

With different THC content, both marijuana (THC > 0.5%) and hemp (THC < 0.3%) belong to *C. sativa* (Chen and Harrington, 2019). In this study, marijuana samples (*N* = 49) were obtained with unknown gender from the Academy of Forensic Science, Ministry of Justice, China, and hemp samples (*N* = 77, 44 males and 33 females) were obtained with known gender from Institute of Bast Fiber Crops, Chinese Academy of Agricultural Sciences. Animal tissue samples (*Canis lupus familiaris*, *Equus caballus*, *Mus musculus*, *Capra hircus*, *Sus scrofa*, *Bos taurus*, *Oryctolagus cuniculus*, *Gallus gallus*, *Anas platyrhynchos*, *Macaque* sp.) and plant tissue samples (*Solanum nigrum*, *Papaver rhoeas*, *Morus alba*, *Salvia japonica*, *Papaver somniferum*, *Humulus lupulus*, and *Humulus scandens*) used for species specificity evaluation were recruited from the Academy of Forensic Science, Ministry of Justice, China. A random high-quality DNA of a female sample in this study was used as a positive control (DM001). Human control DNA 2800M was purchased from Promega (Madison, WI, United States).

All samples were dissected into small pieces with a sterile blade and homogenized using liquid nitrogen. DNA of plant tissue samples was extracted using the DNeasy^®^ Plant Pro Kit (Qiagen, Valencia, CA, United States). DNA of animal tissue samples was extracted using the DNeasy^®^ Blood & Tissue Kit (Qiagen). DNA was quantified by a Qubit dsDNA HS Assay Kit (Invitrogen, Carlsbad, CA, United States).

### Selection of Short Tandem Repeats

Based on previous studies and published reports, the STRs of *C. sativa* were selected as follows: (1) tetranucleotide and trinucleotide repeated STRs were prior to the dinucleotide-repeat STRs. The latter were excluded due to the high stutters and heterozygote imbalances (Valverde et al., 2014b); (2) the STRs with high probability of amplification failures were excluded (Houston et al., 2016; Fett et al., 2019); and (3) all STRs chosen have been previously nominated using the nomenclature of International Union of Pure and Applied Chemistry (IUPAC) (Valverde et al., 2014a,b). In addition, one sex-determining marker and one quality control marker of *C. sativa* from Pei (2009) were also included in the final assay.

### Primer Design and Optimization

DNA sequences of all loci were obtained from the GenBank database (accession number: GCA_900626175.2). PCR primers were designed using Primer Premier v5.0 (PREMIER Biosoft International, Palo Alto, CA, United States) and Oligo v6.0, applying the following main criteria: (1) primer length of 15–30 bp; (2) PCR amplicon length of 50–500 bp; (3) Tm values ranging from 48 to 60°C; (4) Tm values of the forwards and reverse primers at each locus as close as possible; and (5) an optimum GC content ranging from 40 to 60%. The obtained primer pairs were evaluated for non-specific hybridization to other genome regions using the Basic Local Alignment Search Tool (BLAST) in NCBI at http://blast.ncbi.nlm.nih.gov/. Multiplex Manager software v.1.2 was used to check the primer-primer interaction, avoiding potential primer-dimer and hairpin secondary structures.

All selected loci were systematically grouped according to the expected amplicon length and assigned to four different dye-labeling fluorochromes at the 5′ end of 6-FAM (blue), HEX (green), TAMRA (yellow) or ROX (red). All primers were synthesized and labeled by Sangon Biotech Co., Ltd., Shanghai, China.

The amplification of all the developed STRs was performed in a single reaction to evaluate the primer performance and ensure the amplicon size. Each primer was tested with an initial concentration of 0.5 μM. The final concentration of each primer was optimized based on the genotyping results to finally obtain an evenly equilibrated profile.

### Optimization of the Multiplex Polymerase Chain Reaction System

The final optimized multiplex system was performed in a 10 μL reaction volume that included 2.5 μL of 4× PCR Master Mix (PEOPLESPOTINC, Beijing, China), 1.0 μL of 10× Primer Mix, nuclease-free water and 1 ng of template DNA. PCR amplification was carried out using the GeneAmp 9700 PCR system (Applied Biosystems, Foster City, CA, United States).

The annealing temperature in a PCR can affect the specificity of amplification, the yield of products, and the balance of the peaks. Therefore, to minimize the effects of annealing temperature on the multiplex, 1 ng of positive-control DNA (sample #DM001) of *C. sativa* was amplified at different annealing temperature gradients (54, 56, and 58°C) with 28 cycle numbers. In addition, three gradients of PCR cycles (26, 28, and 30) were conducted under the optimized annealing temperature to determine the optimal cycle number of this PCR system. Based on the results of optimization, the final optimum parameters for PCR were as follows: activation at 95°C for 2 min; 28 cycles at 95°C for 5 s, 56°C for 1 min and 60°C for 30 s; and a final extension at 60°C for 5 min. The whole PCR amplification process can finish within 1 h. All PCRs, single and multiplex, included one negative and one positive control.

### Capillary Electrophoresis and Genotyping

The internal size standard was used during CE detection, which is crucial for accurate results of the CE platform. T500 (PEOPLESPOTINC, Beijing, China), which included 19 dye-labeled (“Orange”) DNA fragments (65, 70, 80, 100, 120, 140, 160, 180, 200, 225, 250, 275, 300, 360, 390, 420, 450, 490, and 500 bp) and was selected as the internal size standard for calculating the fragment sizes of PCR products.

For CE progress, the PCR products were subsequently analyzed by adding 1 μL of each amplified product into 9 μL of a 17:1 mixture of Hi-Di formamide (Applied Biosystems, Foster City, CA, United States) and the T500 size standard. The mixture was denatured by heating at 95°C for 3 min and cooling at 4°C for 3 min. Samples were injected electrokinetically at 1.5 kV for 24 s and separated at 15 kV for 1,210 s by a run temperature of 60°C using an ABI 3500*XL* Genetic Analyser (Applied Biosystems, Foster City, CA, United States) and filter set G5 and POP4 polymers (Applied Biosystems, Foster City, CA, United States). The genotyping data of all samples were collected and analyzed using GeneMapper^®^*ID-X* software (Applied Biosystems, Foster City, CA, United States). Allele peaks were set with an analytical threshold of 50 relative fluorescence unit (RFU).

### Preparation of the Allelic Ladder

Allelic ladders were created using a combination of individual templates, which represent the range of alleles observed in the population study. PCR products of different alleles at each locus were cloned into plasmids, and the successful clones of each allele were diluted, mixed, analyzed and balanced to produce a single allelic ladder for each locus (Wang et al., 2014). Those allelic products per locus were mixed and balanced in an appropriate portion to form a “cocktail” (Griffiths et al., 1998). Every allele involved in the in-house ladder was Sanger-sequenced and named according to the nomenclature rules proposed by ISFG (Lincoln, 1997). Based on the length and repeat motif, panel and bin files for GeneMapper^®^ ID-X were programmed. The multiplex system was named the *C. sativa* 19-plex typing system.

### Sizing Precision and Accuracy Study

Sizing precision testing was performed using the developed allelic ladder that was injected on 24 capillaries of the ABI 3500*XL* Genetic Analyser. Subsequently, based on the detailed size information obtained from the injections of the ladder, the average fragment size and standard deviation (SD) of each allele were calculated.

To assess the sizing accuracy, 100 samples were genotyped using the ABI 3500*XL* Genetic Analyser. The sizing accuracy was computed based on the size differences between the alleles of the allelic ladder and the corresponding sample alleles observed from the correct genotypes of each sample.

### Sensitivity Study

To evaluate the sensitivity and optimal amount of DNA input of this multiplex system, serial dilutions of control DNA were amplified with quantities of 2 ng, 1 ng, 500 pg, 250 pg, 125 pg, 62.5 pg, 31.5 pg, and 15.625 pg in triplicate. The percentage of detected alleles and average peak heights were determined for each template DNA.

### Species Specificity Study

To assess the species specificity of this system, 5 ng of non *C. sativa* DNA samples were amplified using the new multiplex, including plant samples (*S. nigrum*, *P. rhoeas*, *M. alba*, *S. japonica*, *P. somniferum*, *H. lupulus*, and *H. scandens*), animal samples (*C. lupus familiaris*, *E. caballus*, *M. musculus*, *C. hircus*, *S. scrofa*, *B. taurus*, *O. cuniculus*, *G. gallus*, *A. platyrhynchos*, *Macaque* sp.), and human control DNA sample 2800M. Each non *C. sativa* DNA sample was detected and analyzed in triplicate to test the cross-reactivity.

### Mixture Study

For the mixture study, the positive control female (DM001) and male (DM030) samples were mixed prior to PCR progress with different ratios (1:1, 1:3, 3:1, 1:9, 9:1, 1:19, and 19:1) and a final amount of 1 ng. Each mixed sample was detected and analyzed in triplicate to test the ability of this system to detect mixtures.

### Stutter Analysis

Stutter peaks are commonly occurring artifacts during the process of PCR. The stutter information was analyzed according to the genotyping profiles obtained from 50 *C. sativa* samples tested on an ABI 3500*XL* Genetic Analyser. Stutters were determined to be peaks with one repeat motif smaller or larger than the true allele. The stutter values were calculated by dividing the peak height of the stutter peaks by the peak height of the true allele. For this study, the analytical threshold of the minimum stutter peak height was set to 20 RFU. Later, the mean stutter value, SD and stutter filter (the mean stutter value plus three SDs) were calculated.

### Balance Analysis

A total of 50 complete profiles were selected, and the balance values were calculated to assess the balance performance (including intralocus balance, intracolour balance and intercolour balance) of the multiplex. Intralocus balance (measuring the balance of heterozygous alleles) was calculated by dividing the height of the smaller peak by the height of the larger peak in a heterozygous pair; intracolour balance (measuring the balance within one color) was calculated by dividing the minimum peak height by the maximum peak height among the same fluorescently labeled loci; intercolour balance (measuring the balance among different colors) was calculated by dividing the minimum peak height by the maximum peak height among all loci regardless of fluorescent label (Zhang et al., 2015).

### Statistical Analysis

To estimate the efficiency and polymorphisms of the included STR markers, a set of 126 *C. sativa* samples (49 marijuana samples and 77 hemp samples) was measured using the *C. sativa* 19-plex typing system. Hardy-Weinberg equilibrium (HWE) and linkage disequilibrium (LD) were calculated by Arlequin v3.5.2 software (Excoffier and Lischer, 2010). PowerStats Version 1.2 software (Promega, Madison, WI, United States) was employed to compute genetic parameters to determine the performance of the STR markers for *C. sativa* analyses, including allele frequencies (AF), observed heterozygosity (Ho), matching probability (MP), power of discrimination (DP), probability of exclusion (PE), and polymorphism information content (PIC). Cumulative discrimination power (CDP) and cumulative probability of exclusion (CPE) were calculated according to the “Specification of paternity testing” issued by the Ministry of Justice, China (SF/ZJD0105001-2016).

## Results and Discussion

### Construction and Optimization of the *Cannabis sativa* 19-Plex Typing System

In this study, we ultimately screened 17 STRs (D02-CANN1, C11-CANN1, 4910, B01-CANN1, E07-CANN1, 9269, B05-CANN1, H06-CANN2, 5159, nH09, ANUCS 501, CS1, ANUCS 305, 3735, ANUCS 302, 1528, and 9043), one sex-determining marker (DM016) and one quality control marker (DM029) in the system. The locus DM016 will be amplified with a fixed length in male samples (marked as “Y”), and the locus DM029 will be amplified with a fixed length in all samples (marked as “1”). To facilitate sex analysis and quality control, the difference between the amplicon sizes of DM016 and DM029 was designed within 15 bp. According to the *C. sativa* reference genome (assembly cs10), we found and presented the detailed chromosomal location of each locus. Except for ANUCS 302 and DM016, which were not found in the reference genome, there were two STRs (ANUCS 501 and 1528) on the X-chromosome (X-STRs) and 15 autosomal STRs (A-STRs) in the *C. sativa* 19-plex typing system. Detailed information on the optimized primers, repeat motifs, size ranges, dye labels, chromosomes and locations are listed in Table 1. Due to the limited development of STR loci of *C. sativa*, five markers on chromosome 4 and three markers on chromosome 2 (with no statistically significant pairwise LD) were selected, which will not affect the efficiency of this system. For ANUCS 302 and DM016, we could infer that they were located on the autosome and Y-chromosome, respectively, because in our study, the alleles of ANUCS 302 showed no difference in distribution between males and females, while DM016 had alleles only in males. Detailed whole-genome sequencing may be needed to define their detailed locations in the future.

**TABLE 1:** General information of 19 STR loci in the *Cannabis sativa* 19-plex typing system.

Marker	Primer sequence (5′-3′)	Repeat motif	Size range (bp)	Primer concentration (μ M)	Dye	Chromosome	Start location of repeat motif	End location of repeat motif
D02-CANN1	F- GGTTGGGATGTTGTTGTTGTG	[GTT]a	93–113	0.03	FAM	2	7798893	7798910
	R- AGAAATCCAAGGTCCTGATGG							
C11-CANN1	F- GTGGTGGTGATGATGATAATGG	[TGA]a[TGG]b	138–180	0.03	FAM	7	6997954	6997986
	R- TGAATTGGTTACGATGGCG							
DM029	F- GATGACAGACTTCCTGATTG	–	219	0.05	FAM	9	7912064	7912291
	R- GTCTAAGAGTGGGAATGCTA							
DM016	F- GCCCAAGTTGCTGCTGAG	–	230	0.03	FAM	Y	/	/
	R- CCCACCGTTTAGGGAGCA							
4910	F- AGATTCCCAAGATGAGCAA	[TTTT]a[TCTT]b	240–300	0.04	FAM	9	50664778	50664821
	R- ACAAACTGGTATCAAGAGCC							
B01-CANN1	F- ATGACATACCAGACAGAAACTC	[TTC]aT[TTC]b	335–460	0.06	FAM	4	34956992	34957040
	R- CATCCATAGCATTATCCCACT							
E07-CANN1	F- CAAATGCCACACCACCTTC	[AGT]a	85–113	0.05	HEX	5	87089686	87089712
	R- GTGGTAGCCAGGTATAGGTAG							
9296	F- CCCAAACTACTGTTTGTGCC	[TTAT]a	114–140	0.04	HEX	4	39968544	39968567
	R- ACTTGCACGTGATGTTAGATCC							
B05-CANN1	F- TTGATGGTGGTGAAACGGC	[TTG]a	217–243	0.03	HEX	6	535865	535882
	R- CCCCAATCTCAATCTCAACCC							
H06-CANN2	F- TGGTTTCAGTGGTCCTCTC	[ACG]a	255–270	0.04	HEX	4	47763149	47763169
	R- ACGTGAGTGATGACACGAG							
5159	F- CCAGAGCTTGTGGATCTCCT	[ATCT]a	312–350	0.04	HEX	8	52664693	52664716
	R- AGTACGAAAGGGCACTGAGG							
nH09	F- CCAACATTTTCTCAGAACCCA	[ATT]a	385–430	0.04	HEX	4	90360758	90360775
	R- TCTTGACTGTAGTAATCCAGC							
ANUCS501	F- AGCAATAATGGAGTGAGTGAAC	[CACAA]a	72–105	0.05	TAMRA	X	21276881	21276900
	R- AGAGATCAAGAAATTGAGATTCC							
CS1	F- AAGCAACTCCAATTCCAGCC	[ATGGTG]a	140–370	0.05	TAMRA	2	92365801	92365902
	R- TAATGATGAGACGAGTGAGAACG							
ANUCS305	F- AGCCCGACCGTGAAGA	[TGA]a[TGG]b [TGA]c	410–450	0.05	TAMRA	2	42415423	42415449
	R- TGAAGCCGATGCCCTAT							
3735	F- TGATTCTGTGTTTGTGTGCAAT	[TATG]a	70–115	0.05	ROX	2	90384783	90384802
	R- CATCGCACCCACAGGTTAGT							
ANUCS302	F- AACATAAACACCAACAACTGC	[ACA]aN6[ACA]5N12	125–165	0.04	ROX	/	/	/
	R- ATGGTTGATGTTTTGATGGT	[ACA]b [CCA]c						
1528	F- GGACTTTGTCTAGTGCCTTTG	[TAAT]a	175–200	0.05	ROX	X	102808838	102808861
	R- GAGTACTTGGCTGATGATGG							
9043	F- AGGTCTGCGTTGTGCATTATT	[TCTT]a	320–355	0.05	ROX	4	177903	177914
	R- AGGGCTGGTTTCAGTTTCG							

*a, b and c represent the repeat number of the repeat motif.*

Initially, several primer pairs of the multiplex were recognized as “failures” at first, which were redesigned and optimized for further use (the primer “failures” is defined as genotyping profiles that exhibit incomplete adenylation, the existence of PCR artefacts, nonspecific products, low fluorescent signal, or no PCR products (Zhang et al., 2014). In addition, for the B01-CANN1 locus, the flanking sequences were highly variable, as observed by Valverde et al. (2014b), resulting in the probability of locus dropout being as high as 9 and 37.5% in Houston et al. (2016) and Fett et al. (2019). Therefore, in the present study, the primers for B01-CANN1 were redesigned, and the amplicon range was expanded. As a result, the amplification success rate of the B01-CANN1 locus was 100% in our 126 samples.

After confirming the successful primers at each locus, all primers were mixed in equal amounts at a concentration of 0.5 μM. Based on the results of genotyping profiles, the concentration of each primer in the multiplex was optimized, and the final concentration of each optimized primer is listed in Table 1.

After testing a range of annealing temperatures, the optimal temperature was selected as 56°C. The average percentages of detected alleles were all 100% at 54, 56, and 58°C. A non-specific peak of ANUCS302 was observed at an annealing temperature of 54°C, and the low amplification efficiency of EO7-CANN1 at 58°C determined the prior annealing temperature to be 56°C. Similarly, the cycle number of PCR was determined to be 28.

**FIGURE 1:**
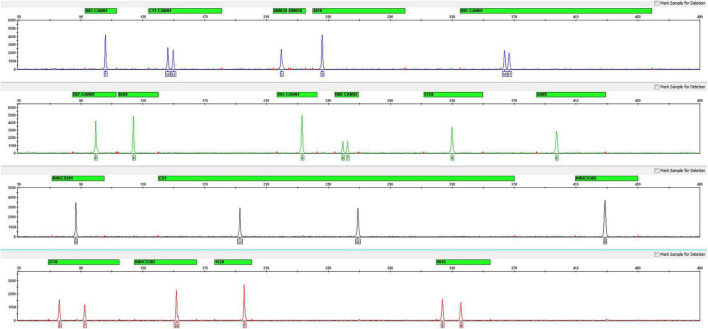
Electropherogram of positive control DNA with the *Cannabis sativa* 19-plex typing system.

After optimizing the PCR conditions of this multiplex, 17 STRs, sex determination and quality control markers were successfully amplified in a single PCR assay. The genotyping profile of the female *C. sativa* DNA (1 ng) is shown in Figure 1, and the male *C. sativa* DNA (1 ng) is shown in Supplementary Figure 1.

### Allelic Ladder

**FIGURE 2:**
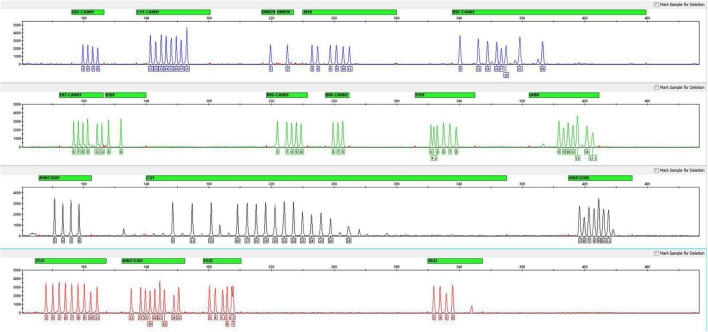
Electropherogram of the allelic ladder designed for the *Cannabis sativa* 19-plex typing system.

For all 17 STRs, one sex-determining marker and one quality control marker, an allelic ladder was developed with the most common alleles observed in the 126 samples. A total of 111 alleles were contained across the 19 loci, and the average peak height of the allelic ladder was 2411 RUF (Figure 2). Despite the sample size limitations, the allelic ladder developed in this study contains the most *C. sativa* alleles observed thus far.

The repeat motif of each allele on each STR involved in the ladder was confirmed by Sanger sequencing and identified by referring to the *C. sativa* reference genome using plus-strand alignment. Notably, the repeat motif of locus ANUCS305 was [TGG]a according to the studies reported by Fett et al. (2019) and Ribeiro et al. (2020). However, when comparing the reference sequence of ANUCS305 (accession number KT203571 in GenBank) with our data, sequence variation was also found on both sides of [TGG]a. For instance, the repeat motif of allele 11 was [TGG]10[TGA], and the repeat motif of allele 12 was [TGA][TGG]10[TGA]. Thus, the repeat motif of ANUCS305 was determined to be [TGA]a[TGG]b[TGA]c in our study.

### Sizing Precision and Accuracy Study

**FIGURE 3:**
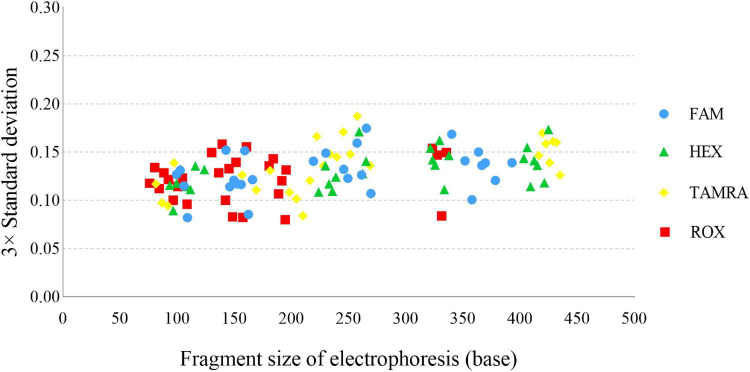
Sizing precision testing across 24 injections of the allelic ladder of the *Cannabis sativa* 19-plex typing system performed on the ABI 3500xl Genetic Analyser.

A sizing precision study is vital for accurate and reliable genotyping, which is assessed by calculating the mean fragment sizes and SD of each allele. In this study, the fragment sizes were plotted against the 3× SD (Figure 3), and the largest SD was only 0.0624 for CS1 at allele 26. We programmed Panel and Bin files based on the above data. For the Bin file, an average allelic peak size of ± 0.5 bp was used as the allele range.

**FIGURE 4:**
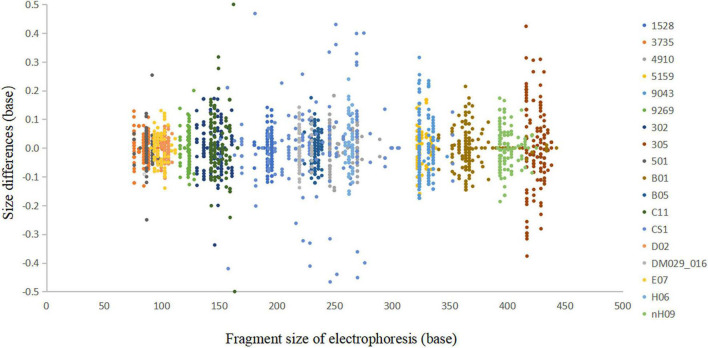
Sizing accuracy study of the *Cannabis sativa* 19-plex typing system performed on the ABI 3500xl Genetic Analyser. These data represent a total of 2,705 alleles from 100 samples.

In the accuracy study, a total of 2,705 alleles from 100 samples were observed within ±0.5 bp of a corresponding allele in an allelic ladder (Figure 4). The results indicated that this multiplex system, together with the homemade allelic ladder and the T500 size standard, was reliable for determining the genotypes and detecting microvariant alleles that differed by one single nucleotide from the original allele.

### Sensitivity Study

For a newly developed genotyping system, it is crucial to evaluate the sensitivity and ability to obtain reliable results from low DNA quantities, as trace DNA samples are common in actual forensic cases. In the sensitivity study, complete genotyping profiles were obtained when the DNA input ranged from 2 ng down to 125 pg, with the average peak heights detected ranging from 4641 RFU to 191 RFU. In addition, when DNA quantities were 62.5 pg, a complete profile was also acquired for one of the three parallel tests with an average peak height of 120 RFU, and only one allele was observed to drop out in the remaining two tests. When the input DNA decreased to 31.25 and 15.625 pg, the percentages of the average loci detected were 79.17 and 68.06%, respectively.

**FIGURE 5:**
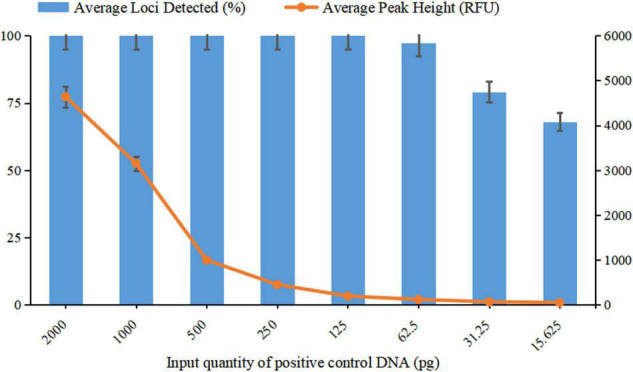
Sensitivity testing of template DNA ranging from 15.625 pg to 2 ng. The average percentage of loci detected was against the DNA input quantity. The left *Y*-axis represents the average percentage of loci detected, and the right *Y*-axis represents the average peak height. Error bars show the SDs between three replicates.

Based on the results of the sensitivity study, the detected peak heights and the average locus detection decreased as the DNA quantity decreased (Figure 5). To avoid allelic dropout or significant heterozygosity imbalance with extremely low DNA quantities or the phenomenon of bleed-through obtained from a DNA template that was too high, the ideal amount of input DNA was suggested to be 125 pg to 2 ng for obtaining high-quality STR profiles from this new system.

### Species Specificity Study

In our study, except for 2800M, *H. lupulus*, *S. japonica*, and *H. scandens*, we did not observe any reproducible peaks above 50 RFU from other non *C. sativa* DNA templates. For 2800M, an off-ladder (OL) peak (peak height: 1514 RFU, size: 419.03 bp) at the B01-CANN1 locus and an OL peak (peak height: 1885 RFU, size: 415.28 bp) at the nH09 locus were detected (Supplementary Figure 2). The results were similar for *H. lupulus*, *S. japonica*, and *H. scandens*, showing several OL peaks or abnormal peaks at D02-CANN1, B01-CANN1, ANUCS305, E07-CANN1, nH09 and 5159 loci (Supplementary Figures 3–5). Although these peaks were observed in the non *C. sativa* DNA, they were either off-ladder or with shapes that were significantly different from the normal peak shape, which would not have any influence on correct genotyping for *C. sativa* DNA. A representative electrophoretogram of animals (*Macaque* sp.) with no reproducible peaks above 50 RFU was also been provided in Supplementary Figure 6.

### Mixture Study

Evidence samples that contain two or more individual *C. sativa* will be encountered in practice (Fett et al., 2019). Thus, it is vital to estimate the ability of this novel 19-plex STR typing system to achieve accurate genotyping data from mixed samples. In this study, the results showed that all the minor alleles of the female (DM001)/male (DM030) mixtures could be detected at the ratios of 1:1 (Supplementary Figure 7), 1:3 and 3:1. In Supplementary Figure 7, 3∼4 alleles were observed at seven STR loci (C11-CANN1, H06-CANN2, 5159, CS1, 3735, ANUCS 302 and 9043), and two imbalanced alleles were found at D02-CANN1, 9269, nH09 and ANUCS501, providing a typical mixed profile from two components. For the mixed ratios of 1:9 and 9:1, an average of 96.67 and 98.04% of the minor alleles were detected, respectively. When the mixture ratio was increased to 1:19 and 19:1, an average of 86.67 and 86.27% of the minor alleles were detected. The average percentage of minor alleles was calculated for each sample across various ratios (Supplementary Figure 8), the results showed a decrease in the percentage of minor alleles that could be identified, as the mixture ratios became higher. In general, these studies indicated that the 19-plex STR typing system has good potential in analyzing mixed samples.

### Stutter Analysis

Stutter peaks are generated by strand slippage during PCR and differ from true peaks by one repeat unit (Brookes et al., 2011). The average stutter ratio and SDs for each STR locus were computed based on the genotyping outputs of 50 samples, which are listed in Table 2. The tetranucleotide-repeat locus 9269 exhibited the lowest average stutter ratio of 0.0051, while the highest average stutter ratio was 0.0557 observed at the trinucleotide-repeat locus B01-CANN1.

**TABLE 2:** Stutter analysis for 17 STR loci of the *Cannabis sativa* 19-plex typing system.

Marker	Min	Max	Mean	SD*^a)^*	Stutter filter*^b)^*
D02-CANN1	0.0027	0.0604	0.0096	0.0098	0.0390
C11-CANN1	0.0105	0.0447	0.0251	0.0092	0.0527
4910	0.0048	0.0650	0.0283	0.0116	0.0631
B01-CANN1	0.0052	0.0820	**0.0557**	0.0224	0.1229
E07-CANN1	0.0201	0.1075	0.0498	0.0243	0.1227
9269	0.0027	0.0083	**0.0051**	0.0013	0.0090
B05-CANN1	0.0054	0.0551	0.0229	0.0121	0.0592
H06-CANN2	0.0027	0.0521	0.0081	0.0113	0.0420
5159	0.0035	0.0224	0.0062	0.0036	0.0169
nH09	0.0051	0.1202	0.0228	0.0300	0.1129
ANUCS501	0.0036	0.0162	0.0058	0.0032	0.0155
CS1	0.0089	0.0537	0.0245	0.0119	0.0601
ANUCS305	0.0040	0.0472	0.0232	0.0169	0.0738
3735	0.0059	0.0624	0.0240	0.0158	0.0713
ANUCS302	0.0141	0.2174	0.0358	0.0398	0.1551
1528	0.0020	0.0288	0.0068	0.0059	0.0244
9043	0.0079	0.0163	0.0111	0.0038	0.0226

*Stutter values were obtained from the genotyping data of 50 samples. The analytical threshold of the minimum stutter peak height was set to 20 RFU.*

*^a^SD, standard deviation.*

*^b^Stutter filter = Mean + 3 × SD (shown as decimal value).*

### Balance Analysis

Three parameters for balance studies (intralocus balance, intracolour balance and intercolour balance) were calculated using 50 samples by the *C. sativa* 19-plex typing system. As shown in Supplementary Table 1, the values for intralocus balance ranged from 0.8095 (CS1) to 0.9516 (4910), and the values for intracolour balance ranged from 0.6191 (HEX) to 0.6809 (TAMRA). For the intercolour balance, the value was 0.7072. To ensure accurate heterozygote genotyping and the detection of low template or degraded samples, the intralocus balance, intracolour balance and intercolour balance are recommended to be greater than 0.7, 0.5, and 0.3, respectively (Collins et al., 2004). All data obtained for these three balance values of the *C. sativa* 19-plex typing system satisfied the established standards. In summary, the system we developed presented highly balanced performance.

### Statistical Analysis

Different sexes of *C. sativa* contain different amounts of THC, and only female plants have medicinal value and even abuse potential, making it very important to identify the sex of *C. sativa*. Morphologically, the sex of *C. sativa* can only be determined during flowering; thus, a fast and reliable biological method for identifying the sex of *C. sativa* is urgently needed. In the present study, we added the *C. sativa* sex-determining marker DM016 to the new system, which performed sex identification on 126 samples. Among the 126 *C. sativa* samples, 44 were males and 82 were females; 49 marijuana samples were all females, and the sex-determining results of 77 hemp (44 males and 33 females) were consistent with known genders. Based on the current data and previous studies (Dembiński et al., 2008), it stands to reason that all marijuana are females, while further verification is needed using more marijuana samples. To the best of our knowledge, this is the first publication involving a *C. sativa* sex-determining marker in the STR multiplex.

Aforementioned 126 samples of *C. sativa* were amplified using the *C. sativa* 19-plex typing system, and 120 complete DNA profiles were successfully obtained. However, six marijuana samples suffered single locus dropout at nH09 (3.2%) and ANUCS 305 (1.6%). The electropherograms showed that the peak heights decreased with increasing amplicon size until nH09 and ANUC S305 (the amplicon fragments were larger than 400 bp) dropped out. Furthermore, single PCRs for nH09 and ANUCS305 were conducted for the above 6 samples, and no amplification peaks were generated, which excluded the cause of primer-primer interactions with the dropouts. Then, we reviewed that the six marijuana samples were old materials from real cases, indicating that the DNA degradation of these samples might therefore result in locus dropout. Intriguingly, ANUCS 501 is located on the X chromosome, but it still showed heterozygous genotypes in 20 male samples, so we speculated that ANUCS 501 is located in the recombination region between the X- and Y-chromosomes.

To achieve an ideal *C. sativa* group with unrelated samples, we removed samples collected from the same case (retaining one sample per case) and ended up with 85 *C. sativa* samples (27 marijuana and 58 hemp). A total of 162 alleles at 15 A-STRs and 14 alleles at 2 X-STRs were obtained from 85 samples, while H06-CANN2 had the least number of variants with three alleles, and CS1 had the greatest number of variants with 35 alleles. The *p*-value of HWE was significant for nine STR loci (*p* < 0.05) (Table 3), and similar results were observed by Ribeiro et al. (2020). The main factors of the deviations from HWE in many loci could be explained by asexual reproduction of *C. sativa* and the sampling bias in this study; the latter could be improved by expanding the sample size in further studies. In addition, no statistically significant pairwise LD was detected between the 15 A-STRs after applying Bonferroni’s correction, except for that between ANUCS305 and H06-CANN2. However, ANUCS305 and H06-CANN2 are located on different chromosomes, and previous studies have shown that the two loci had no deviations from LD (Houston et al., 2016). Therefore, the genetic frequencies of all 15 A-STRs were subsequently used to calculate CDP and CPE.

**TABLE 3:** Parameters for 15 autosomal STRs in *Cannabis sativa* (*N* = 85).

Marker	Allele number	Ho	MP	PIC	PD	PE	*p*-HWE
D02-CANN1	4	0.5647	0.2938	0.4554	0.7062	0.2506	0.8221
C11-CANN1	8	0.6353	0.0940	0.7278	0.9060	0.3354	0.1044
4910	10	0.6353	0.1421	0.6241	0.8579	0.3354	0.5878
B01-CANN1	27	0.8118	0.0425	0.8492	0.9575	0.6211	0.0284*
E07-CANN1	9	0.6118	0.1391	0.6664	0.8609	0.3052	0.0261*
9269	5	0.3177	0.4419	0.3430	0.5581	0.0711	0.0200*
B05-CANN1	5	0.5882	0.1787	0.5815	0.8213	0.2770	0.2955
H06-CANN2	3	0.3882	0.3412	0.3978	0.6588	0.1069	0.0277*
5159	8	0.4941	0.2720	0.4902	0.7280	0.1824	0.3110
nH09	13	0.5059	0.0807	0.7800	0.9193	0.1927	0.0000*
CS1	35	0.8588	0.0154	0.9455	0.9846	0.7123	0.0341*
ANUCS 305	9	0.5529	0.0685	0.8067	0.9315	0.2382	0.0000*
3735	10	0.7765	0.0588	0.8127	0.9412	0.5561	0.0269*
ANUCS 302	10	0.6471	0.0812	0.7720	0.9188	0.3512	0.0010*
9043	6	0.6000	0.1704	0.6009	0.8296	0.2909	0.2823

*Significant p-value.

Based on the genotyping data, allele frequencies and parameters for each locus were calculated (Table 3). The Ho values varied from 0.3430 (9269) to 0.8588 (CS1), with an average Ho of 0.5992. The PIC values ranged from 0.2754 (9269) to 0.9455 (CS1), with an average PIC of 0.6569. The DP values for most STRs were above 0.7, with the exception of 9269 (0.5581) and H06-CANN2 (0.6588), and the average DP value was 0.8386. The PE values ranged from 0.0711 (9269) to 0.7123 (CS1), with an average value of 0.3218. In this study, CS1 was found to be the most informative maker, and similar results were observed in Houston et al. (2016), Fett et al. (2019), and Ribeiro et al. (2020). In total, the TDP and the CPE of the 19-plex typing system were calculated to exceed 0.999 999 999 999 988 and 0.998 455 889 684 078, respectively, presenting higher efficiency than two multiplex panels containing 13-STR markers (Fett et al., 2019; Ribeiro et al., 2020).

## Conclusion

This study described the development and validation of a fast detection method for *C. sativa*, the 19-plex STR typing system; 15 autosomal STRs (D02-CANN1, C11-CANN1, 4910, B01-CANN1, E07-CANN1, 9269, B05-CANN1, H06-CANN2, 5159, nH09, CS1, ANUCS 305, 3735, ANUCS 302 and 9043), two X-chromosome STRs (ANUCS 501 and 1528), one sex-determining marker (DM016) and one quality control marker (DM029) were included in the 5-dye multiplex, in which all loci were co-amplified in a single PCR system within 1 h, which was suitable for fast detection of *C. sativa*. Following the guidelines issued by SWGDAM and ISFG, validation studies of the new system were carried out and indicated that the 19-plex typing system was accurate, sensitive and *C. sativa*-specific. Meanwhile, the new system also showed superior discriminative power and a high combined paternity exclusion probability value, successfully achieving the aims of species identification, gender determination, and individual identification of the *C. sativa*. Thus, this system could be a useful tool for police in tracing back trade routes of particular drug syndicates or dealers and linking a certain *C. sativa* to a crime scene. Additionally, we first reported the chromosomal location of the STRs and sex-determining markers for *C. sativa*, which facilitates further studies concerning *C. sativa*. Due to the limited development of STR loci of *C. sativa*, several STRs on a same chromosome were selected in this study, while it would be more informative to include more markers that cover all the chromosomes of *C. sativa.* Currently, we are developing polymorphism STR loci on different chromosomes of *C. sativa*, which will provide more informative STRs in the future.

## Data Availability Statement

The raw data presented in the study are included in the article/Supplementary Material, further inquiries will be made available by the authors, without undue reservation.

## Ethics Statement

The animal study was reviewed and approved by the Ethics Committee of the Academy of Forensic Sciences, Ministry of Justice, China.

## Author Contributions

CL and SZ conceived the study and supervised the whole project. RX and RT drafted the main manuscript text and made the data analysis. YQ and XZ conducted the experiment. HY and CY helped to advise the manuscript. All authors read and approved the final manuscript.

## Conflict of Interest

The authors declare that the research was conducted in the absence of any commercial or financial relationships that could be construed as a potential conflict of interest.

## Publisher’s Note

All claims expressed in this article are solely those of the authors and do not necessarily represent those of their affiliated organizations, or those of the publisher, the editors and the reviewers. Any product that may be evaluated in this article, or claim that may be made by its manufacturer, is not guaranteed or endorsed by the publisher.

## Supplementary Material

Supplementary Figure 1 Electropherogram of the male control DNA with the *Cannabis sativa* 19-plex typing system.

Supplementary Figure 2 Electropherogram of the human control DNA (2800M) with the *Cannabis sativa* 19-plex typing system.

Supplementary Figure 3 Electropherogram of *Humulus lupulus* DNA with the *Cannabis sativa* 19-plex typing system.

Supplementary Figure 4 Electropherogram of *Salvia japonica* DNA with the *Cannabis sativa* 19-plex typing system.

Supplementary Figure 5 Electropherogram of *Humulus scandens* DNA with the *Cannabis sativa* 19-plex typing system.

Supplementary Figure 6 Electropherogram of *Macaque* sp. DNA with the *Cannabis sativa* 19-plex typing system.

Supplementary Figure 7 Electropherogram of the mixture DM001:DM030 at the ratios of 1:1 with the *Cannabis sativa* 19-plex typing system.

Supplementary Figure 8 The mixture DM001:DM030 was explored with serial mixed ratios. The average percentages of the detected minor alleles vs. the different mixed ratios are shown, and a full genotyping profile could be achieved at the ratios of 1:1, 1:3, and 3:1.
