# Supplementary material for: Development and Validation of a Novel and Fast Detection Method for Cannabis sativa: A 19-Plex Short Tandem Repeat Typing System
Source: Front Plant Sci. 2022 Feb 28;13:837945. doi: 10.3389/fpls.2022.837945 (PMC8918947; doi:10.3389/fpls.2022.837945)
Supplement: Supplementary file 1 [file Table_1.DOCX]

**Table S1.** Balance calculation for the 19-plex STR typing system. Three parameters (a. Intra-locus, b. Intra-color and c. Inter-color) were calculated based on genotyping results of 50 samples. For the intra-locus parameter, 17 STR loci were analyzed (the balance criteria: intra-locus balance above 0.7, intra-color balance above 0.5 and inter-color balance above 0.3).

|  | Min | Max | Mean | SD^a)^ |
| --- | --- | --- | --- | --- |
| **a. Intra-locus** |  |  |  |  |
| D02-CANN1 | 0.7663 | 0.9936 | 0.9113 | 0.0677 |
| C11-CANN1 | 0.5874 | 0.9866 | 0.8691 | 0.1201 |
| 4910 | 0.8874 | 0.9942 | 0.9516 | 0.039 |
| B01-CANN1 | 0.5998 | 0.9805 | 0.8688 | 0.1063 |
| E07-CANN1 | 0.6137 | 0.9872 | 0.9066 | 0.0975 |
| 9269 | 0.8439 | 0.9948 | 0.9259 | 0.0556 |
| B05-CANN1 | 0.8309 | 0.9921 | 0.9202 | 0.0506 |
| H06-CANN2 | 0.7614 | 0.9977 | 0.9196 | 0.0747 |
| 5159 | 0.6692 | 0.9972 | 0.8632 | 0.1148 |
| nH09 | 0.4239 | 0.9997 | 0.8495 | 0.1348 |
| ANUCS501 | 0.7744 | 0.9934 | 0.9225 | 0.0728 |
| CS1 | 0.5392 | 0.9914 | 0.8095 | 0.1232 |
| ANUCS305 | 0.7706 | 0.9896 | 0.9071 | 0.0637 |
| 3735 | 0.7375 | 0.9976 | 0.9357 | 0.0636 |
| ANUCS302 | 0.8203 | 0.9988 | 0.904 | 0.0612 |
| 1528 | 0.8286 | 0.9955 | 0.917 | 0.0634 |
| 9043 | 0.8209 | 0.9931 | 0.9351 | 0.0507 |
| **b. Intra-color** |  |  |  |  |
| FAM | 0.4731 | 0.9763 | 0.6391 | 0.0971 |
| HEX | 0.4873 | 0.9599 | 0.6191 | 0.1068 |
| TAMRA | 0.4675 | 0.9118 | 0.6809 | 0.1327 |
| ROX | 0.4465 | 0.8294 | 0.6759 | 0.0954 |
| **c. Inter-color** | 0.5034 | 0.8811 | 0.7072 | 0.0873 |

a) SD: standard deviation
