# Supplementary figures and images for: Development and Validation of a Novel and Fast Detection Method for Cannabis sativa: A 19-Plex Short Tandem Repeat Typing System
Source: Front Plant Sci. 2022 Feb 28;13:837945. doi: 10.3389/fpls.2022.837945 (PMC8918947; doi:10.3389/fpls.2022.837945)

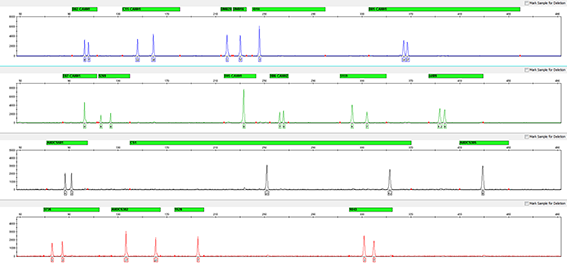

Supplement: Supplementary Figure 1 — Electropherogram of the male control DNA with the Cannabis sativa 19-plex typing system. [file Image_1.TIF]

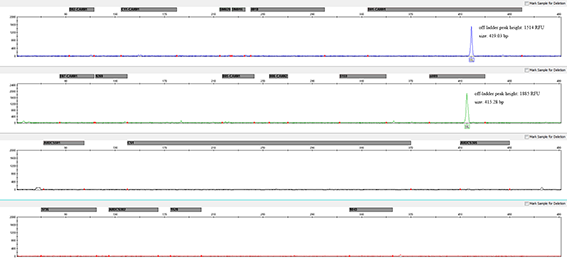

Supplement: Supplementary Figure 2 — Electropherogram of the human control DNA (2800M) with the Cannabis sativa 19-plex typing system. [file Image_2.TIF]

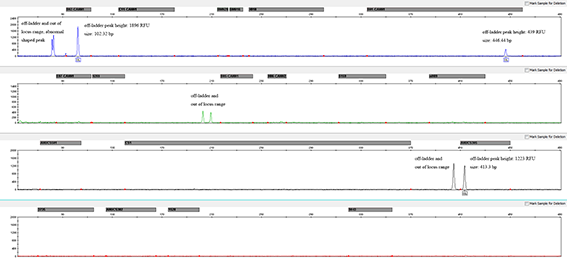

Supplement: Supplementary Figure 3 — Electropherogram of Humulus lupulus DNA with the Cannabis sativa 19-plex typing system. [file Image_3.TIF]

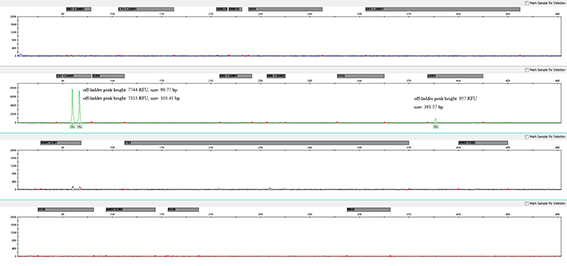

Supplement: Supplementary Figure 4 — Electropherogram of Salvia japonica DNA with the Cannabis sativa 19-plex typing system. [file Image_4.TIF]

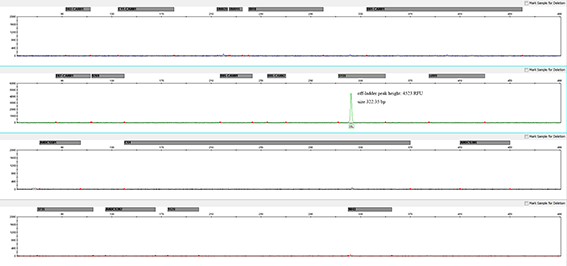

Supplement: Supplementary Figure 5 — Electropherogram of Humulus scandens DNA with the Cannabis sativa 19-plex typing system. [file Image_5.TIF]

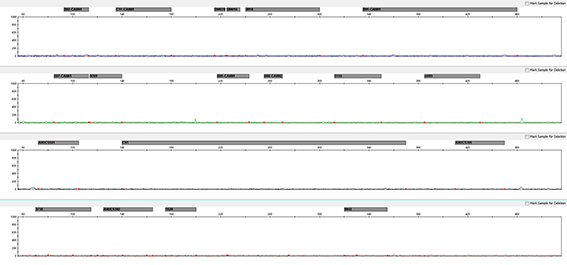

Supplement: Supplementary Figure 6 — Electropherogram of Macaque sp. DNA with the Cannabis sativa 19-plex typing system. [file Image_6.TIF]

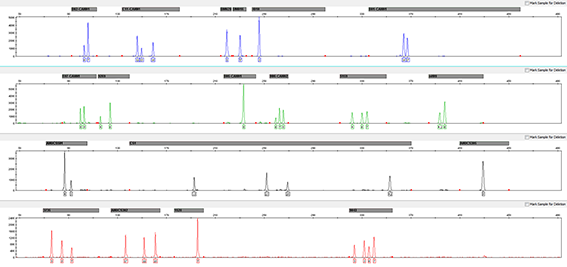

Supplement: Supplementary Figure 7 — Electropherogram of the mixture DM001:DM030 at the ratios of 1:1 with the Cannabis sativa 19-plex typing system. [file Image_7.TIF]

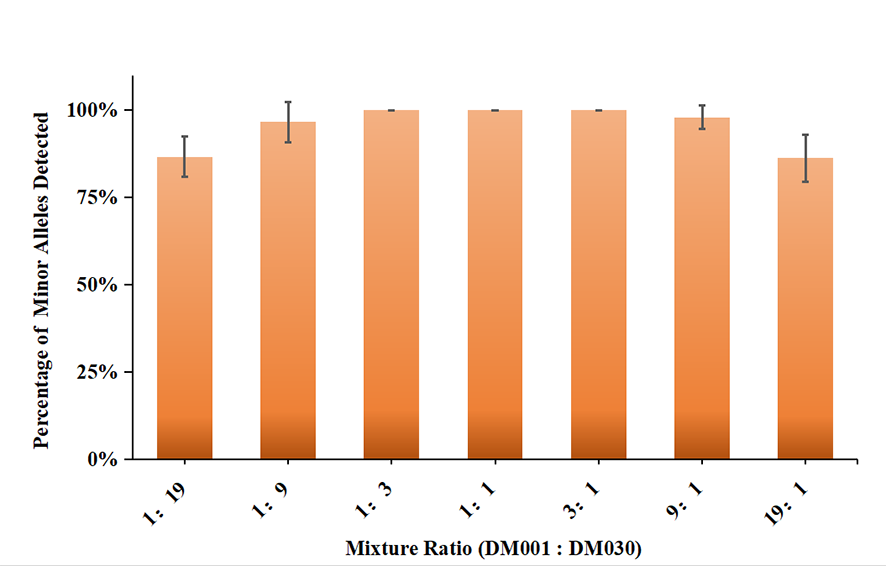

Supplement: Supplementary Figure 8 — The mixture DM001:DM030 was explored with serial mixed ratios. The average percentages of the detected minor alleles vs. the different mixed ratios are shown, and a full genotyping profile could be achieved at the ratios of 1:1, 1:3, and 3:1. [file Image_8.TIF]
